# Aorta-sparing total arterial redo-CABG using a recycled patent LIMA: a consecutive 24-patient series with midterm clinical and computed tomographic outcomes

**DOI:** 10.1186/s13019-026-04543-6

**Published:** 2026-07-16

**Authors:** Ahmad Ali Amirghofran, Alireza Arzhangzadeh, Soorena Khorshidi, Salma Nozhat, Javad Kojuri, Mahsa Borjzadehgashtaseb, Mohammad Rafati Navaei

**Affiliations:** 1 Department of Cardiac Surgery, Shiraz University of Medical Sciences, Shiraz, Iran; 2 Department of Cardiology, Shiraz University of Medical Sciences, Shiraz, Iran

**Keywords:** Redo-CABG, LIMA reuse, total arterial revascularization, coronary artery disease, graft patency

## Abstract

**Background:**

Redo coronary artery bypass grafting (Redo-CABG) poses significant challenges due to complex coronary anatomy and limited conduit availability. In patients with a previously patent left internal mammary artery (LIMA), reusing it as an inflow source for total arterial revascularization (TAR) may offer a durable, stroke-sparing strategy. This study evaluated the safety and midterm outcomes of reusing a patent LIMA as an inflow source in Redo-CABG.

**Methods:**

This retrospective case series included 24 patients who underwent Redo-CABG at Faghihi and Dena Hospitals (Shiraz University of Medical Sciences) between March 2015 and October 2023. Consecutive patients were included to minimize selection bias. All patients had a patent LIMA confirmed by angiography or computed tomography angiography (CTA). Arterial grafts (right internal mammary and/or radial artery) were anastomosed to the patent LIMA to achieve complete TAR. Outcomes included perioperative complications, graft patency on CTA, and midterm major adverse cardiac or cerebrovascular events (MACCE).

**Results:**

All patients achieved complete TAR with no hospital or 30-day mortality. One patient required reoperation for bleeding. Mean postoperative ejection fraction was 44.5 ± 4.6%, and mean ICU stay was 3.1 ± 0.9 days. Early postoperative CT angiography (at 3 months) and midterm follow-up imaging (median 3.5 years; completed in a subgroup of 12 patients) demonstrated complete graft patency in all evaluated conduits, with no recorded MACCE.

**Conclusions:**

Reusing a patent LIMA as an inflow source for total arterial grafting in Redo-CABG appears to be a feasible and safe alternative for high-risk patients with limited conduit options. This approach minimizes extensive aortic manipulation, potentially offering a theoretical advantage in minimizing neurological risks. However, the retrospective, single-center design and small sample size preclude definitive conclusions regarding superiority, warranting further comparative evaluation in larger multicenter studies.

**Supplementary Information:**

The online version contains supplementary material available at https://doi.org/10.1186/s13019-026-04543-6.

## Introduction

Coronary artery disease (CAD) remains one of the leading causes of morbidity and mortality worldwide [1]. Despite advances in percutaneous interventions and pharmacologic therapy, coronary artery bypass grafting (CABG) continues to offer the most durable revascularization in multivessel disease [2]. Re-operative coronary artery bypass grafting (Redo-CABG) is being performed more infrequently in contemporary practice, accounting for approximately 2% of all CABG procedures worldwide, reflecting changing trends in incidence and evolving patient profiles [3]. However, approximately 5–10% of patients require repeat revascularization within 10 years, often due to graft failure, disease progression, or new lesions in native vessels [4]. Redo-CABG is technically demanding and carries higher perioperative risk compared to primary surgery. Patients often present with diffuse coronary lesions, poor target vessels, and limited conduit options [5]. Saphenous vein grafts (SVGs), commonly used in the initial operation, often degenerate over time and are frequently unavailable for reuse [6]. In addition, reoperative procedures carry increased risks of aortic manipulation, including embolic stroke, due to atheromatous disease of the ascending aorta [7]. These challenges highlight the need for strategies that provide complete and durable myocardial revascularization with minimal procedural risk.

Total arterial revascularization (TAR) using internal thoracic and radial arteries has emerged as a superior strategy, providing enhanced long-term graft patency, improved survival, and reduced adverse cardiac events compared to traditional saphenous vein graft–based approaches [8, 9]. The left internal mammary artery (LIMA) to the left anterior descending artery (LAD) remains the gold standard of coronary grafting, with patency exceeding 95% at 10 years [10]. When a LIMA graft from a previous CABG remains patent, it can potentially serve as a reliable inflow source for additional arterial conduits, allowing full revascularization without proximal aortic anastomoses [11]. Previous small series have reported that reusing a patent internal mammary artery during Redo-CABG is feasible and safe, with acceptable midterm outcomes [12, 13]. Evidence remains limited, largely because most available studies are retrospective and describe selective rather than complete arterial revascularization. The present study aimed to evaluate the feasibility, safety, and midterm outcomes of reusing an old patent LIMA as the inflow source for total arterial revascularization in Redo-CABG, exploring its potential as an aorta-sparing alternative for patients with limited conduit options.

## Methods

### Patient selection and eligibility criteria

**Fig. 1:**
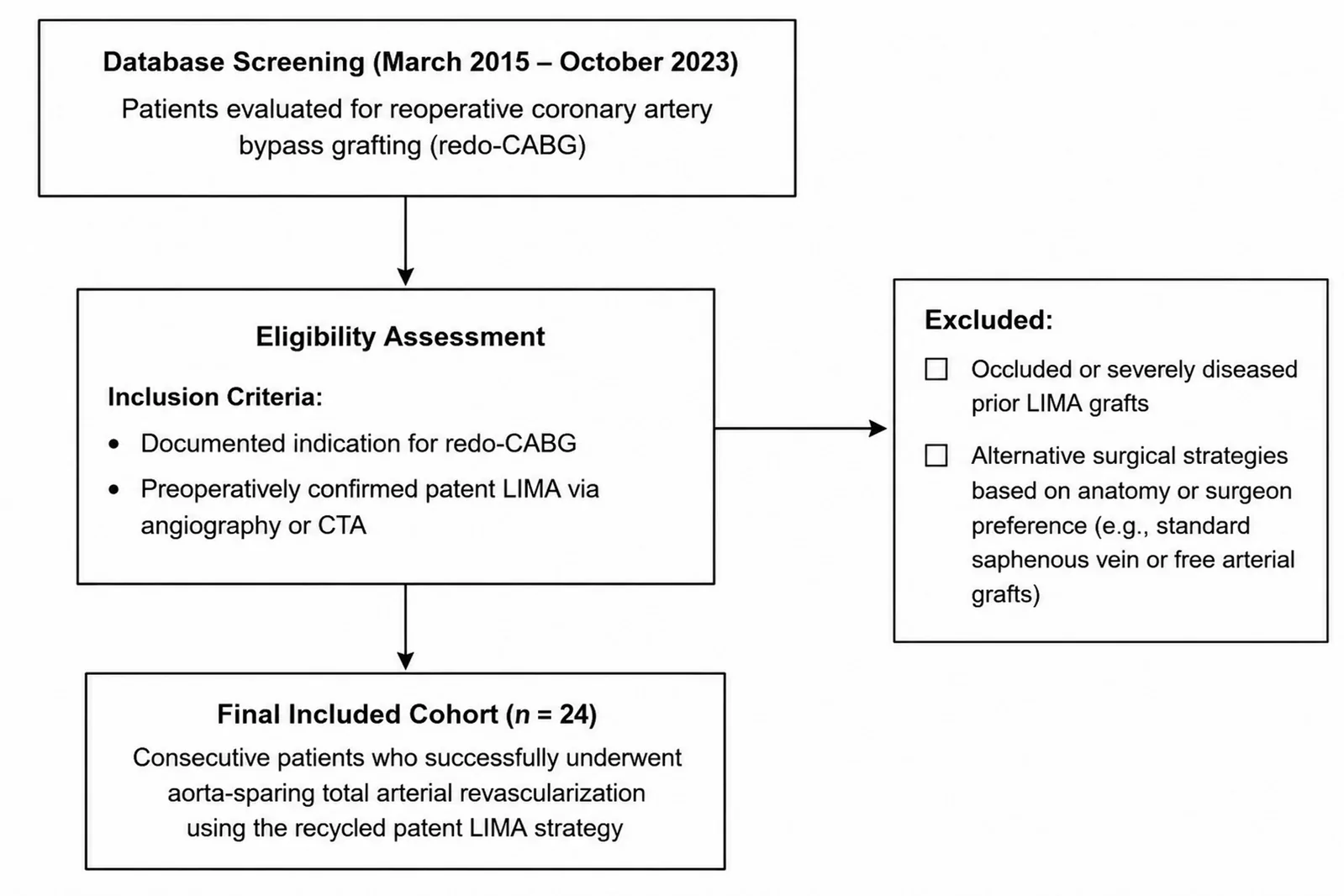
Patient Selection and Screening Pathway Diagram. Flowchart demonstrating the institutional database screening criteria, inclusion protocol, and final consecutive cohort selection

Patients were screened from the institutional cardiothoracic surgery database covering the period between March 2015 and October 2023. The screening pathway was strictly based on objective anatomical and clinical eligibility. All consecutive patients who presented with a documented indication for Redo-CABG and met the core inclusion criteria—specifically, an angiographically or CT-confirmed patent LIMA graft from the primary surgery and a surgical decision to employ the LIMA-reuse strategy—were included in this series. Patients presenting with an occluded or severely diseased prior LIMA graft, or those who underwent conventional Redo-CABG via standard alternative conduits (such as isolated saphenous vein or free arterial grafts) due to surgeon preference or anatomical suitability, were excluded from this specific cohort. Ultimately, 24 consecutive patients fulfilled the complete eligibility protocol and underwent the procedure. To maximize methodological transparency, the screening and selection pathway is illustrated in Fig. 1.

### Preoperative assessment and imaging protocol

All patients underwent detailed preoperative assessment, including echocardiography, coronary angiography, and coronary CT angiography. CT imaging was specifically used to evaluate the patency, diameter, and anatomical course of the old LIMA relative to the sternum to minimize the risk of conduit injury during resternotomy.

### Surgical technique and composite graft configurations

Resternotomy was performed using an oscillating saw, with meticulous adhesiolysis under direct vision. The orientation of the previously grafted LIMA was reviewed preoperatively to facilitate safe exposure. The patent LIMA was gently mobilized to an adequate length while preserving its blood supply.

In cases with open LIMA–LAD anastomoses, the LIMA was partially dissected and temporarily clamped using a bulldog clamp before cardioplegia administration and aortic cross-clamping. Cardiopulmonary bypass (CPB) was initiated with standard cannulation. Custodiol cardioplegia was used in all cases due to its long-acting myocardial protection, which is advantageous in redo procedures.

Additional arterial conduits—right internal mammary artery (RIMA) and/or radial artery—were harvested and used to achieve total arterial revascularization. Depending on target vessel anatomy and conduit length, various composite configurations were constructed, including T-graft, Y-graft, or sequential graft techniques. When necessary, limited proximal anastomoses were performed on the aorta, though this was avoided whenever feasible. Transit-time flow measurement (TTFM) was used intraoperatively to verify flow adequacy and pulsatility index for all grafts.

Following hemostasis, temporary pacing wires and chest drains were inserted. Patients were then transferred to the intensive care unit (ICU) for postoperative care and monitoring.

### Postoperative care and clinical follow-up protocols

Postoperative management followed institutional protocols. Early graft patency was assessed by CTA at 3 months, and midterm follow-up was performed at a median of 3.5 years. The primary endpoint was graft patency confirmed by CTA. Secondary endpoints included 30-day mortality, major adverse cardiac or cerebrovascular events (MACCE), left ventricular ejection fraction (EF), and functional recovery based on New York Heart Association (NYHA) classification.

### Data management and statistical analysis

Demographic, perioperative, and outcome data were collected using predefined forms. Continuous variables were expressed as mean ± standard deviation (SD), and categorical variables as frequency and percentage. Normality was evaluated using the Shapiro–Wilk test. Statistical analyses were performed using SPSS version 26.0 (IBM Corp., Armonk, NY, USA).

## Results

### Patient characteristics

A total of 24 patients underwent redo coronary artery bypass grafting (CABG) using a previously patent left internal mammary artery (LIMA) as an inflow conduit. The mean age was 60.4 ± 10.7 years, and 79% were male. The median interval between primary and Redo-CABG was 12 years (interquartile range 9–15 years). Diabetes mellitus was the most prevalent comorbidity (87.5%), followed by hypertension (71%) and hyperlipidemia (29%). The majority of patients (87%) were in NYHA functional class II before surgery, with a mean preoperative ejection fraction (EF) of 46.5 ± 6.5%.

Table 1 summarizes the demographic and preoperative clinical characteristics.

**Table 1:** Demographic and preoperative clinical features of patients undergoing Redo-CABG

Variable		Value
Age (years); mean ± SD		60.40 ± 10.73
Gender	Male	19 (79%)
	Female	5 (21%)
Comorbid disease	DM	21 (87.5%)
	HTN	17 (71%)
	HLP	7 (29%)
Smoking history	Positive	6 (25%)
	Negative	18 (75%)
Previous CABG Time in years; mean ± SD		12.20 ± 4.56
Number of Previous CABG Grafts; number (%)	2	1 (4%)
	3	11 (46%)
	4	10 (42%)
	5	2 (8%)
Occluded grafts; number (%)	1	2 (8%)
	2	12 (50%)
	3	9 (38%)
	4	1 (4%)
NYHA Classification; number (%)	2	21 (87%)
	3	3 (13%)
Previous EF (%); mean ± SD	46.5 ± 6.5	

SD: Standard deviation; CABG: Coronary artery bypass graft; DM: Diabetes mellitus; EF: Ejection fraction; HLP: Hyperlipidemia; HTN: Hypertension; NYHA: New York Heart Association

### Intraoperative findings

All procedures were performed on-pump using Custodiol cardioplegia. Mean cardiopulmonary bypass (CPB) time was 104.2 ± 25.9 min, and mean aortic cross-clamp time was 80.1 ± 14.6 min. Each patient achieved complete total arterial revascularization (TAR) using the patent LIMA as an inflow source. The right internal mammary artery (RIMA) and radial artery were used in all cases, and various composite graft configurations were constructed according to target vessel distribution.

The mean number of grafts per patient was 3.9 ± 0.6. The mean number of grafts connected to the old patent LIMA was 2.5 ± 0.7. Transit-time flow measurement (TTFM) demonstrated satisfactory intraoperative flows (mean graft flow 38 mL/min, mean pulsatility index 2.4). Table 2 presents intraoperative variables.

**Table 2:** Intraoperative features of patients undergoing Redo-CABG

Variable		Value
CPB time (minutes); mean ± SD		104.2 ± 25.9
Aortic clamp time (minutes); mean ± SD		80.10 ± 14.60
Cardioplegia; n (%)	Custodiol	24 (100%)
On CPB HCT (%); mean ± SD		29.40 ± 3.42
Number of arterial grafts in current operation; n (%) or mean ± SD	Average	3.9 ± 0.6
	3	4 (17%)
	4	18 (75%)
	5	2 (8%)
Number of grafts on old LIMA in current operation; n (%) or mean ±SD	Average	2.5 ± 0.7
	1	2 (8%)
	2	7 (29%)
	3	15 (63%)
RIMA use; n (%)		24 (100%)
Radial use; n (%) *		24 (100%)
Artery used on old LIMA	Rad	7 (29%)
	RIMA	13 (54%)
	RIMA + Rad	4 (17%)
Number of composite grafts; n (%)	2	6 (25%)
	3	16 (67%)
	4	2 (8%)
Intra-aortic balloon pump; n (%)	Yes	1 (4%)
	No	23 (96%)
Mean Graft Flow via TTFM (mL/min); mean ± SD		38.0 ± 7.4
Mean Pulsatility Index (PI) via TTFM; mean ± SD		2.4 ± 0.5

SD: Standard deviation; CPB: Cardiopulmonary Bypass; ACC: Aortic Cross-Clamp; LIMA: Left Internal Mammary Artery; TTFM: Transit-Time Flow Measurement; HCT: Hematocrit; LIMA: Left internal mammary artery; RIMA: Right internal mammary artery; Rad: Radial artery* The radial artery was used in 100% of cases, either directly anastomosed to the LIMA or in other configurations

### Postoperative outcomes

All 24 operations were completed successfully without in-hospital or 30-day mortality (0%; 95% confidence interval [CI]: 0.0%–14.2%). One patient required reoperation due to postoperative bleeding. The mean ICU stay was 3.1 ± 0.9 days, and mean postoperative EF was 44.5 ± 4.6%. Eighteen patients (75%) required epinephrine infusion, and four (17%) required norepinephrine support. One patient required intra-aortic balloon pump insertion, and one developed transient contrast-induced nephropathy that resolved with conservative management. Table 3 summarizes postoperative outcomes.

**Table 3:** Post-operative features of patients undergoing redo-CABG

Variable			Value
Extubation time (hours); mean ± SD			11.2 ± 5.0
Epinephrine*		Frequency; number of patients who required epinephrine infusion as an inotrope; n (%)	18 (75%)
		Infusion time (for those 18 patients) (hours); mean ± SD	16.05 ± 11.78
ICU Blood Transfusion (ml); mean ± SD			530 ± 371.1
Bleeding volume in ICU (ml); mean ± SD			391.5 ± 391.0
Reoperation in ICU; n (%)		Yes	1 (4%)
		No	23 (96%)
ICU stay (days); mean ± SD			3.1 ± 0.9
Post-operation EF (%); mean ± SD			44.5 ± 4.6
30-day MACCE	Yes		0 (0%) [95% CI: 0.0%–14.2%]
	No		24 (100%)
Contrast-Induced Nephropathy (CIN)	Yes		1 (4%)
	No		23 (96%)

SD: Standard deviation; ICU: Intensive Care Unit; EF: Ejection Fraction; IABP: Intra-Aortic Balloon Pump; CIN: Contrast-Induced Nephropathy; MACCE: Major Adverse Cardiac and Cerebrovascular Events. *The less post-operative Epinephrine infusion time the less rate of hospital mortality and renal dysfunction in patients undergoing cardiac surgery based on Shahin .et al research (20)undefined

### Graft patency and follow-up

**Fig. 2:**
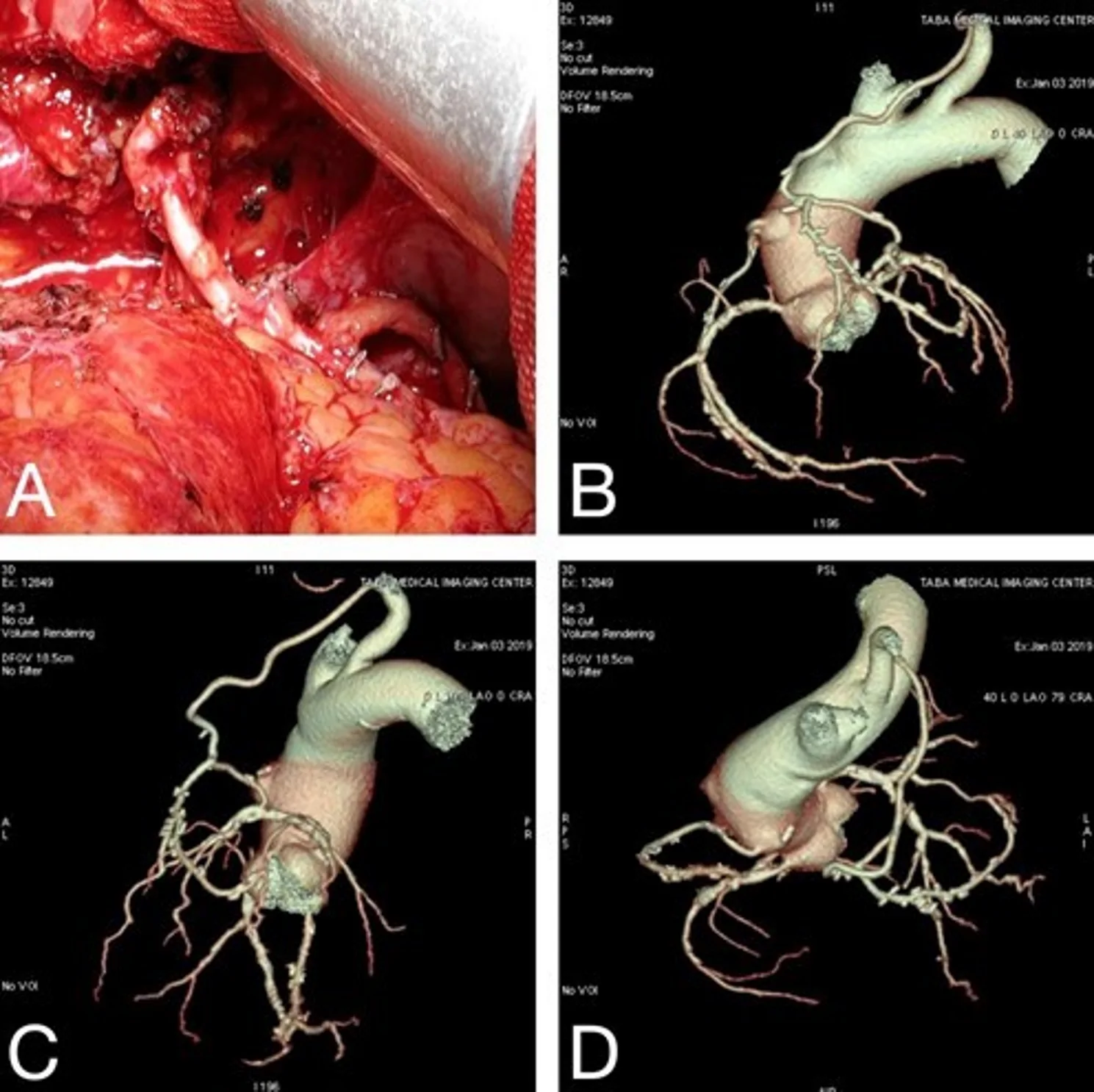
LIMA T-graft configuration: intraoperative image and 3-month 3D CTA follow-up. **A**: Intraoperative image showing the left internal mammary artery (LIMA) after adhesiolysis. A secondary arterial conduit has been anastomosed to the LIMA in a T-graft configuration (white arrow indicates LIMA inflow source; yellow arrow indicates composite branch outflow). **B–D**: Different views of three-dimensional CTA reconstructions at 3-month follow-up in the same patient, demonstrating the early anatomical configuration, luminal patency, and architectural distribution of the LIMA and its T-graft extension from various anatomical perspectives”

The midterm clinical follow-up milestone (median 3.5 years) was completed for the entire cohort of 24 patients (100% clinical compliance) through outpatient clinical visits or structured telephone interviews to monitor long-term clinical events. Regarding the objective imaging protocol, early postoperative CTA (at 3 months) was successfully performed in all patients. At the midterm follow-up milestone, repeat CTA was successfully completed in a subgroup of 12 patients who consented to repeat radiological evaluation or presented with clinical indications. These assessments demonstrated complete graft patency in all evaluated conduits at their respective imaging timepoints. A representative example of LIMA-based T-graft configuration and its early postoperative CTA findings is shown in Fig. 2. No imaging follow-up was performed for the remaining asymptomatic patients due to geographical distance, lack of clinical indications, or patient refusal. No patient experienced myocardial infarction, stroke, or other MACCE during the follow-up period (0%; 95% CI: 0.0%–14.2%). Mean follow-up left ventricular ejection fraction remained stable compared with baseline. Table 4 summarizes operative configurations and grafting techniques used, while the detailed individual data of the operative techniques are compiled in Supplementary Table 1.

**Table 4:** Operative configurations and grafting techniques (summary)

Configuration type	Description	Frequency (%)
LIMA inflow with RIMA composite (Y/T grafts)	RIMA and radial arteries attached to old LIMA inflow for multivessel TAR	100%
Sequential radial grafts on LIMA	Used for OM and PDA territories	71%
Mixed RIMA + radial configurations	Combined Y and sequential arrangements	29%
Aortic anastomosis required	Limited cases when conduit length insufficient	12%

## Discussion

This study demonstrates that reusing a patent left internal mammary artery (LIMA) as an inflow conduit for total arterial revascularization (TAR) during redo coronary artery bypass grafting (CABG) is both feasible and safe. In this single-university experience involving 24 patients, complete arterial revascularization was achieved in all cases, with no mortality, no major adverse cardiac or cerebrovascular events (MACCE), and complete graft patency confirmed by CTA at midterm follow-up. Additionally, none of the patients experienced recurrent angina during follow-up, further supporting the clinical success of this surgical strategy; however, it should be noted that these patients did not present with acute coronary syndrome, which may limit the generalizability of this finding.

### Rationale for LIMA reuse

The excellent hemodynamic performance of the recycled LIMA can be mechanistically attributed to its high basal nitric oxide production and preservation of endothelial integrity, which accommodates the increased flow demand of composite branches. However, potential failure modes of this strategy must be considered, including competitive flow from native coronary vessels, surgical trauma to the fragile old LIMA during resternotomy, or localized steal syndrome if the inflow diameter is borderline. Conceptually, compared to using free arterial or venous grafts, this approach preserves the natural retrosternal geometry and minimizes proximal aortic anastomoses. Furthermore, aortic manipulation in redo operations increases the risk of embolic stroke and bleeding [6, 7]. In this context, an all-arterial approach that avoids the ascending aorta is particularly advantageous.

The LIMA has consistently demonstrated superior long-term patency, with reported rates exceeding 95% at 10 years. Its unique histologic features—including a thin media, rich nitric oxide production, and resistance to atherosclerosis—make it an ideal conduit for sustained myocardial perfusion. When the previously grafted LIMA remains patent, it offers a valuable and safe source of inflow for additional arterial grafts, especially when other conduits are exhausted [10]. The TAR-CABG population had better long-term survival at 18.65 years compared with radial ± vein grafting without IMA [14].

### Comparison with previous studies

Our findings align with and expand upon previous reports describing the feasibility of internal mammary artery (IMA) reuse. El Oumeiri et al. (2011) reported 60 patients who underwent Redo-CABG using recycled IMAs, with an 8.3% 30-day mortality and satisfactory midterm survival [15]. Noirhomme et al. (1999) documented favorable outcomes in 16 patients, though angiographic follow-up was limited [16]. Pasic et al. (2005) described zero mortality and excellent graft patency in 12 patients [17], while Agrifoglio et al. (2007) confirmed long-term freedom from restenosis of 75% over 15 years [18].

Compared with these studies, our series represents one of the larger homogeneous case series achieving complete total arterial revascularization using a patent LIMA inflow, with standardized imaging follow-up confirming complete graft patency. A meta-analysis of 23 studies (*n* = 100,314) with mean follow-up of 8.8 years showed reduced all-cause mortality for TAR (hazard ratio < 1) compared to nontotal arterial strategies [9]. In our cohort, no perioperative mortality or MACCE occurred, which benchmarks favorably against standard Redo-CABG series where perioperative mortality often ranges from 2% to 7% in contemporary registries. These findings support the technical reliability of this approach when performed with careful preoperative planning.

### Technical considerations

Mobilization of a previously grafted LIMA requires careful preoperative assessment and intraoperative handling. Off-pump total arterial grafting in elderly patients did not demonstrate a significant survival or MACCE-free advantage over 5 years in comparative cohorts [19].

Preoperative CT angiography is crucial to determine the graft’s position relative to the sternum and to plan a safe resternotomy. During surgery, partial dissection and temporary clamping of the LIMA may be required to avoid ischemia and ensure safe cardioplegia administration.

Various composite graft configurations—Y, T, or sequential—can be constructed using the right internal mammary artery (RIMA) and radial artery to achieve complete arterial coverage. In our experience, the hemodynamic performance of these grafts was excellent, with satisfactory transit-time flow measurements (mean graft flow 38 mL/min; mean pulsatility index 2.4). These findings confirm that an old patent LIMA can provide adequate flow to multiple territories when used as the main inflow conduit.

### Advantages and clinical implications

The principal advantages of this approach include:

1. Theoretical reduction of stroke risk via avoidance of extensive ascending aortic manipulation.
2. Preservation of arterial-only revascularization, ensuring optimal long-term durability.
3. Feasibility in patients with limited conduit options, particularly those with previous SVG harvesting or peripheral vascular disease.
4. Excellent hemodynamic performance, confirmed intraoperatively by TTFM and radiographically by CT angiography.

This ‘last shot, best shot’ strategy allows surgeons to provide patients with the most durable configuration during their final opportunity for surgical revascularization.

### Limitations

This study shares limitations common to previous single-institution retrospective series. The relatively small sample size limits statistical power and generalizability, and selection bias cannot be excluded. The absence of a control group precludes direct comparison with conventional redo-CABG using vein or free arterial grafts. Although the median follow-up of 3.5 years showed excellent patency, longer-term surveillance is required to confirm durability beyond a decade. Consequently, our findings represent clinical feasibility and association rather than definitive therapeutic superiority. The reported excellent outcomes may partly reflect careful patient selection and institutional expertise, meaning that conclusions regarding effectiveness should be interpreted with caution. Furthermore, systematic imaging follow-up was not uniform across all time points for the entire cohort. While clinical follow-up was 100% complete, midterm CTA was only obtained in a subset of 12 patients. This imaging attrition introduces a potential survival or outcome verification bias, as asymptomatic patients were less likely to undergo repeat radiological evaluations at midterm.

## Conclusion

Reusing a patent left internal mammary artery as an inflow conduit in redo coronary artery bypass grafting represents a feasible surgical strategy with encouraging midterm imaging-confirmed patency in carefully selected patients. While this aorta-sparing approach conceptually reduces the need for ascending aortic manipulation, further larger comparative trials are required to definitively establish its long-term durability and clinical advantages over conventional reoperative techniques.

## Supplementary Information

Below is the link to the electronic supplementary material.

Supplementary Material 1.

## Data Availability

No datasets were generated or analysed during the current study.

## Abbreviations

CABG: Coronary artery bypass grafting
CAD: Coronary artery disease
PCI: Percutaneous coronary intervention
TAR: Total arterial revascularization
MACCE: Major adverse cardiac or cerebrovascular events
SVG: Saphenous vein graft
CTA: Computed tomography angiography
TTFM: Transit-time flow measurement
LIMA: Left internal mammary artery
RIMA: Right internal mammary artery
