# Supplementary material for: Aorta-sparing total arterial redo-CABG using a recycled patent LIMA: a consecutive 24-patient series with midterm clinical and computed tomographic outcomes
Source: J Cardiothorac Surg. 2026 Jul 16;21:756. doi: 10.1186/s13019-026-04543-6 (PMC13656523; doi:10.1186/s13019-026-04543-6)
Supplement: Supplementary file 1 [file 13019_2026_4543_MOESM1_ESM.docx]

**Supplementary Table 1: Detailed Patient-Level Target Anatomy, Prior Graft Status, and Reoperative Surgical Configurations**

| Patient No. | Primary operation | Reoperative procedure |
| --- | --- | --- |
| 1 | LIMA-LAD (patent LAD diseased) / SVG-Ramus (severe stenosis)/ SVG-PDA (severe stenosis) | RIMA-LAD distal (connected to old patent LIMA) / SVG-OM1 / Radial PDA, PLVB (connected to mid part RIMA) |
| 2 | Only LIMA patent | RIMA-distal LAD (free graft to old LIMA)/ Radial-OM1 (T-graft on old LIMA)/ Radial OM2 (-T- graft on o d LIMA)/ RIMA-PLVB (free graft to PDA)/ Radial –PDA On Aorta) |
| 3 | LIMA-LAD (LAD stenosis) / SVG-OM (occluded) */* SVG-PDA (occluded) | RIMA-OM (T-graft on previous LIMA)/ RIMA-LAD (arterial patch on localized stenosis)/ Radial -PDA (direct to aorta) |
| 4 | *LIMA-LAD (patent LIMA, anastomosis site occluded)* | RIMA Distal LAD (free graft to old LIMA)/ RIMA-D1 (T-graft) / RIMA-D2/ Radial-OM2 (direct to aorta) |
| 5 | LIMA-LAD (post-graft LAD stenosis) / SVG-D1 (occluded) | RIMA-LAD (previous old LIMA)/ Radial-OM1 (previous old LIMA)/ Radial-PDA (previous old LIMA) sequential |
| 6 | LIMA-LAD (patent, stenotic LAD)/ SVG-D1 (occluded) / SVG­OM1 (occluded) / SVG OM2 (occluded)/ SVG PDA (patent, stenotic) | RIMA-LAD (Y-graft old LIMA)/ Radial-OM1 (T-graft old LIMA)/ Radial OM2 (T-graft old LIMA) / SVG- RCA |
| 7 | Only LIMA LAD patent | Radial-OM1 (T-graft on LIMA)/ Radial-D1 (sequential) / Radial-RCA (RCA endarterectomy free graft)/ RIMA-PDA (after endarterectomy) |
| 8 | LIMA-LAD patent/ SVG-OM1, OM2, OM3, RCA Occluded | RIMA-OM1 (T-graft LIMA)/ RIMA-OM2 (T-graft LIMA)/ RIMA-OM3 (T-graft LIMA)/ Radial-PDA (aorta) |
| 9 | All previous grafts occluded/ LIMA-LAD patent | RIMA-LAD (connected to old LIMA)/ RIMA-OM1 (T-graft RIMA­LIMA)/ RIMA-D1 (T-graft RIMA-LIMA)/ Radial-PLVB (aorta) |
| 10 | LIMA-LAD patent | RIMA-RCA / Radial-D1 (connected to LIMA)/ Radial-OM1 (connected to LIMA)/ Radial-OM2 (connected to LIMA) |
| 11 | LIMA LAD patent/ SVG-OM1, OM2, RCA Occluded | RIMA-OM2 (connected to LIMA)/ RIMA-OM3 (sequential)/ Radial-PDA (aorta) |
| 12 | LIMA-LAD Patent/ SVG-D2 stenotic / SVG-RCA occluded | RIMA-OM1 (free graft to LIMA)/ RIMA-OM2 (sequential)/ Radial­-D1 (connected to LIMA)/ Radial-RCA (aorta) |
| 13 | LIMA-LAD patent, Stenosis after anastomosis/ SVG-OM occluded/ SVG-PDA occluded | RIMA-LAD (old LIMA distal LAD)/ RIMA-OM1 (old LIMA)/ Radial-PDA (aorta)/ Radial -PLVB (sequential) |
| 14 | LIMA-LAD patent severe stenosis after anastomosis/ All SVGs occluded | Radial-LAD (connected to LIMA)/ Radial-PDA end (sequential)/ RIMA-OM2 (connected to radial) |
| 15 | LIMA-LAD patent/ SVG-PDA patent/ SVG-OM occluded | RIMA-Distal LAD (connected to top end SVG)/ Radial-D1 (LIMA) / Radial-OM1 (sequential)/ Radial-OM2 (sequential) |
| 16 | LIMA-LAD patent / SVG-PDA, OM1, OM2 Occluded | RIMA-D1 (T-graft LIMA) / SVG-OM1 / SVG-OM2 (sequential) / Radial-RCA, aorta via SVG to end / Radial-PDA (sequential) |
| 17 | LIMA-LAD patent / SVG-PLVB patent / SVG-D1 occluded/ SVG-OM1 occluded | RIMA-LIMA(T-graft)-D1-OM1(sequential)/ Radial-PDA on SVG cap (PLVB) |
| 18 | LIMA-LAD patent/ SVG-PDA occluded/ SVG-OM2 occluded/ SVG-D2 occluded | Radial-LIMA(T-graft)-D2 then on OM2(sequential)/RIMA-Radial (Y-graft) on PDA |
| 19 | LIMA-LAD patent significant mid LAD stenosis / SVG-D1 occluded/ SVG-OM1 occluded | RIMA as arterial patch on LAD stenosis/ RIMA-LIMA(T-graft) on D1 then OM1 (sequential) /Radial-PDA (direct on aorta) |
| 20 | LIMA-LAD patent/ SVG-PDA occluded/ SVG-OM2 occluded | RIMA-LIMA (Y-graft)- distal LAD, RIMA-LIMA(T-graft)-OM2 /Radial-PDA (direct on aorta) |
| 21 | LIMA-LAD patent/ SVG-PDA occluded/ SVG-D1 occluded/ SVG-OM1 occluded | Radial -LIMA (T-graft)-D1 then sequential-OM1 /RIMA on Radial (Y graft)-PDA |
| 22 | LIMA-LAD patent/ SVG-RCA occluded/ SVG-OM1 occluded | RIMA-LIMA(T-graft)-OM1 then OM2(sequential) /Radial-PDA (direct on aorta) |
| 23 | LIMA-LAD patent/ SVG-PDA patent/ SVG-OM1 occluded/ SVG-D1 occluded | Radial-LIMA(T-graft)-D1-OM1(sequential) /RIMA-Radial (Y-graft) then on PLVB |
| 24 | LIMA-LAD patent, distal LAD stenosis/ SVG-OM1 occluded/ SVG-PDA occluded/ SVG-D2 occluded | RIMA-LIMA(T-graft)-D2/ RIMA-LIMA (Y-graft) to distal LAD / Radial -RIMA (Y-graft)-OM1 then PDA (sequential) |
